# Analysing healthcare coordination using translational mobilization

**DOI:** 10.1108/JHOM-05-2017-0116

**Published:** 2018-05-21

**Authors:** Davina Allen

**Affiliations:** Department of Healthcare Sciences, Cardiff University, Cardiff, UK

**Keywords:** Service quality, Implementation, Improvement, Coordination, Organization-of-care, Translational mobilization theory

## Abstract

**Purpose:**

The purpose of this paper is to introduce translational mobilization theory (TMT) and explore its application for healthcare quality improvement purposes.

**Design/methodology/approach:**

TMT is a generic sociological theory that explains how projects of collective action are progressed in complex organizational contexts. This paper introduces TMT, outlines its ontological assumptions and core components, and explores its potential value for quality improvement using rescue trajectories as an illustrative case.

**Findings:**

TMT has value for understanding coordination and collaboration in healthcare. Inviting a radical reconceptualization of healthcare organization, its potential applications include: mapping healthcare processes, understanding the role of artifacts in healthcare work, analyzing the relationship between content, context and implementation, program theory development and providing a comparative framework for supporting cross-sector learning.

**Originality/value:**

Poor coordination and collaboration are well-recognized weaknesses in modern healthcare systems and represent important risks to quality and safety. While the organization and delivery of healthcare has been widely studied, and there is an extensive literature on team and inter-professional working, we lack readily accessible theoretical frameworks for analyzing collaborative work practices. TMT addresses this gap in understanding.

## Introduction

Poor coordination and collaboration are well-recognized weaknesses in modern healthcare systems and represent important risks to quality and safety (Institute of Medicine, 2001). While the organization and delivery of healthcare has been widely studied, and there is an extensive literature on team and inter-professional working, we lack readily accessible theoretical frameworks for analyzing collaborative work practices (Greenhalgh, 2008). Translational mobilization theory (TMT) is a new framework that addresses this gap in understanding (Allen and May, 2017). This paper introduces TMT and explores its potential value for analyzing the coordination of collective action in healthcare for quality improvement purposes.

## The challenge of coordination in healthcare

There is a growing realization that high quality healthcare depends not on individual brilliance, but on making sure that all the elements required to meet patient needs – people, expertise, materials, technologies and information – are aligned. “Right care. Right place. Right time” may be the Holy Grail of quality improvement, but this is not easy to achieve and the consequences of getting this wrong are serious. A recent Canadian hospital’s institution-wide mortality review reported that the most important quality gaps in this organization arose from the failure of healthcare workers to align their efforts around two key goals: treatment plans and diagnosis (Kobewka *et al.*, 2016).

The challenges of coordination in healthcare are well known and arise from three sources. First, healthcare is a work of “many hands” (Aveling *et al.*, 2016); patients receive input from different healthcare providers and specialists and these relationships are conditioned by differences in knowledge, occupational culture, social worlds, power and prestige. Furthermore, while there is much use of the language of teamwork in this field, in reality every day service provision is characterized by action and knowledge that is distributed across time and space, fragmented and multiple understandings of the patient, and staff that make largely independent contributions to care (Allen, 2015). Second, this complex system of work is embedded in an inherently turbulent environment. Healthcare organizations have rather less control over their inputs and outputs than do other services and industries and experience constant churn (Duffield *et al.*, 2007). Thus, the care of individual patients has to be balanced with the care of whole populations and providers have to manage competing demands on their time. Third, healthcare is unavoidably “people work” and thus has qualities that are not present when the object of work is inanimate (Strauss *et al.*, 1985). Individual’s care can evolve in unpredictable directions – which is increasingly common in an aging population with multiple health conditions and care needs – and of course, patients and their families have a view on these processes too: they are both producers and consumers of healthcare (Osborne *et al.*, 2013). These features of healthcare work pose very real challenges for care coordination, which, according to Strauss *et al.*, mirror those of Mark Twain’s celebrated Mississippi River pilot:

[…] the river was tricky, changed it’s course slightly from day-to-day, so even an experienced, but inattentive pilot could run into grave difficulties; worse yet, sometimes the river drastically shifted in its bed for some miles into a new course. […] Some of the various contingencies may be anticipated, but only a portion of them may be relatively controllable […] stemming as they do, not only from the illnesses themselves but from organizational sources. In some instances, contingencies may also stem from sources external to the hospital

(Strauss *et al.* 1985, pp. 19-20).

Strauss *et al.* (1985) captured this dynamic in the notion of an illness trajectory defined as “the physiological unfolding of a patients’ disease” and “the total organization of work done over that course, plus the impact on those involved with that work and its organization.” The challenges of coordination are understood as arising not only from the uncertainty of attending to injury and disease, but also from the complexity of the division of labor and the turbulence of the work environment. While some trajectories are more or less foreseeable and can be managed through generic coordination mechanisms, in many others the mobilization of action depends less on rational plans and structures, than it does on people working together in emergent patterns of organization in response to contingencies.

## Theorizing collaboration in emergent organizations

Healthcare is a quintessential example of the challenges of organization in complex and turbulent environments, but in other contexts there is evidence that bureaucratic models (Gerth and Mills, 1946) are increasingly being replaced by emergent and networked organizational forms (Castells, 2009). Empirical examples include: offshore software development (Boden *et al.*, 2008), film production (Bechky, 2003), global engineering (Pernille and Christensen, 2011) and marketing (Kellogg *et al.*, 2006). We lack readily available theoretical frameworks for understanding collective action of this kind, the result of an historical legacy in which research on organizational systems and structures – and their relations with their environments – became decoupled from investigations into the things that people in organizations actually do (Barley and Kunda, 2001; Dingwall, 2015). Despite recent conceptual and methodological advances in fields such as computer supported cooperative work and practice-based approaches in organizational studies (Nicolini, 2009), these have not generated a coherent theoretical framework for analyzing the coordination and organization of collaborative activity. TMT aims to address this gap in understanding.

TMT is a generic sociological theory that was developed to support the analysis of collective action wherever this is found. The purpose of this paper is to introduce the theory and how it facilitates the understanding collective action specifically in healthcare where it has potential utility for quality improvement purposes. A full account of the theory and its theoretical and empirical foundations can be found in Allen and May (2017).

## TMT

### Foundational assumptions

TMT belongs to a family of approaches known as practice theories (Nicolini, 2013) and directs attention to the socio-material practices through which social life is accomplished. It is based on four core assumptions about the organization of work that it is important to understand when applying the theory. First, it adopts an ecological approach to cooperative activity (Hughes, 1984). In the same way in which the concept of the ecosystem in biology underlines the network of interactions between organisms in a particular locale, ecological approaches to collective action underscore the dynamic and emergent qualities of systems of work and the inter-relationships between people, materials and technologies within a given context. Second, and following on from this, TMT takes a process view of formal organizations and underlines the agency of the people who work in them. Seemingly durable social forms, that is, structures or institutions, such as professional roles and organizational routines, are conceptualized as on-going accomplishments that come into being through their use in action. The aim here is to capture the relationship between the active and interpretative dimensions of everyday life, whilst recognizing that the world appears as external to and a constraint on action. Third, while foregrounding human agency, TMT also deploys insights from activity theory (Engeström, 2000) on the role of artifacts in mediating action. It draws attention to the fact that we never interact directly with the social world; all activity is mediated through artifacts of some kind. This might include material artifacts, such as tools, technologies and instruments, or cognitive artifacts, such as categories, heuristics and methods. Artifacts are the means through which participants in systems of work create and understand the objects of their practice – whether this is a patient, an engineering project or a product to be marketed – and they condition the possibilities for action. This leads to the fourth assumption of TMT, which is to conceptualize collaborative work as distributed not only between people, but also across materials and technologies. This insight is derived from actor network theory (ANT) and directs attention to the relationships between people, materials and technologies in a field of practice and how these are distributed in time and space (Latour, 1995, 2005). These core elements form the domain assumptions of TMT and together offer a rather different conceptualization of healthcare work than is typical in quality improvement initiatives (Box I).

### Core components

TMT comprises three core components: the project (the focus of action and “what” is done), the strategic action field (“where” it is done) and the mechanisms of mobilization and institutionalization (“how” it is done) (see Table I).

For quality improvement purposes these provide a framework for analyzing projects of collective action and the conditions and actions necessary to embed improvements into practice.

Box I **Conceptualizing healthcare work with TMT**

Formal organization: in healthcare, the formal components of organizations and institutions – such as work roles, rules or routines – are typically treated as external drivers of human activity. Within TMT, social institutions and human agency are conceptualized as working back and forth in a dynamic relationship. Thus while institutions shape the possibilities for action, their meanings are enacted by human agents through their use in practice. This invites us to understand the normal condition of healthcare organization as an on-going tension between stability and fluidity, formality and informality, as members make sense of and deploy the available social structures and resources in carrying out their work within a given organizational context. The dynamic of this relationship can have positive or negative effects. Ethnographic studies of innovation in healthcare highlight how interventions become embedded in organizations because of the negotiations, workarounds and tinkering work that people do to integrate them into practice for patient benefit (Timmermans and Berg, 2003). Conversely, this relationship can lead to normalized deviance and have a negative impact on healthcare quality (Barach and Phelps, 2013). Understanding this relationship and its implications for healthcare quality should be at the center of any improvement effort and current debates about implementation and sustainability.

Patients: in healthcare, patients are conventionally treated as if they have pre-existing and stable identities, with care coordination believed to depend on patient centered approaches and good communication. Without wishing to deny the human dimensions of care, TMT draws attention to the multiple versions of the patient *created* by healthcare systems, as patients are understood in relation to a wide range of practices, technologies and methodologies. Considered in this way, as the objects of healthcare practice, “patients” have to be understood as decentered, that is, distributed in social time and space between services, providers and technologies. For example, Mol (2002) has shown how the construction of a patient with atherosclerosis that is observed in the clinic, is different from that which is displayed in the vascular laboratory and different again from the picture that emerges in the operating theater. In a similar vein, Taee (2017) describes how patients in Bhutan are understood in different ways by orthodox, traditional and alternative approaches to medicine as they seek cures for their ailments. Acknowledging the spread and multiplicity of patient identities in a collaborative endeavor raises critical questions about the understanding necessary to enable collective action and how this is achieved.

Socio-material practices: TMT focuses attention on the social, material and cognitive processes through which healthcare activities are accomplished and in particular how action is mediated by artifacts. In healthcare, for example, multiple artifacts are deployed in the work of patient care. These include formal artifacts such as diagnostic categories, risk assessment scores, laboratory tests. It also includes informal artifacts, such as the heuristics, technologies and methodologies individuals deploy to support their practice. Examples include the diagnostic rules of thumb used by ENT surgeons and found to be responsible for widely different rates of tonsillectomies (Bloor, 1976), the “mind lines” deployed by general practitioners (Gabbay and Le May, 2004), or the “scraps” deployed by nurses to manage their patient care work (Hardey *et al.*, 2000). TMT takes seriously the actions that are accomplished through such socio-material arrangements and how these mediate action in different contexts. Understanding that artifacts do not simply support work activity, they transform the nature of the task and redistribute work, highlights how the introduction of new artifacts has important consequences for practice (Allen, 2012, 2016). Even so, despite the proliferation of artifact use in improvement efforts, they receive scant attention in implementation processes (Allen, 2017).

The following section illustrates the core components of TMT taking the example of a rescue trajectory as an illustrative case. A rescue trajectory refers to the collective action involved in detecting and acting upon signs of deterioration in hospitalized patients where coordination and collaboration across professional and departmental boundaries in conditions that are emergent and uncertain is critical for success. Failure to detect and act on signs of deterioration is an acknowledged safety concern in healthcare (Subbe and Welch, 2013).

### The application of TMT for healthcare improvement: the case of rescue trajectories

#### The project

The project is the primary unit of analysis in TMT and directs attention to the collective activity identified as in need of change. For improvement purposes, the “project” provides a frame for the analysis of the ecological relationships in a collective activity. Depending on the purpose, in some instances it may be helpful to consider a project in the round, and in others there could be value in dividing a project into sub-projects. In the case of a rescue trajectory, beyond the overall process from detection of deterioration to intervention, one might focus on key trajectory components – such as, monitoring and recording, interpretation of information, review and initiation of action, response and intervention – and their inter-relationships. An alternative strategy would be to take an individual actor – such as an observation chart or an occupational role – and consider the wider project of activity in which it is located. Understanding this situated context (Allen, 2013) can be of particular value for implementation purposes as it brings into view the wider network of relationships into which a technology is embedded and the implications this has for normalization. Projects are often intertwined (Engeström, 2000), and it may be necessary to consider intersecting projects or lines of work (Strauss, 1988), particularly if these have the potential to affect or be affected by planned changes to practice. In our example, this might prompt the improvement team to ask questions about other activities that interact with rescue trajectories – such as recovery pathways from surgical procedures, the organization of nursing care or pain management.

#### Strategic action field

The importance of understanding context for research and quality improvement purposes is well established (The Health Foundation, 2014; May *et al.*, 2016). In TMT, the strategic action field (Fligstein and McAdam, 2011) refers to the institutional context in which projects are progressed and provides a framework to support systematic consideration of the salient features that condition projects of social action and which are likely to be consequential for the success or failure of an intervention.

This includes attending to the social structures implicated in a project and how the network of relationships – roles, status, organizations, departments, teams, and hierarchies – mediate social action. In the case of a rescue trajectory this would include a particular focus on the division of labor between nursing and support staff (in some contexts, healthcare assistants are responsible for undertaking monitoring activity, whereas in others, this is a nursing responsibility); inter-professional relationships, particularly the relationship between nursing and medicine (mobilizing action across professional boundaries/hierarchies is critical for timely intervention in rescue trajectories and is known to be challenging) (Andrews and Waterman, 2005); and relationships between the ward or unit and emergency response team (we know that escalation decision making is influenced by interaction at this interface) (Cioffi, 2000).

Organizing logics are those elements of a strategic action field that drive action and define a project’s scope and purpose. While at one level the higher order aim of a rescue trajectory should be the same in any organization – to detect and act on signs of deterioration – these may be mediated by local conditions. For example, the drivers for action and organizational logics in a tertiary hospital with collocated intensive care and high dependency units will be quite different from those of a smaller hospital with no such facilities and where escalation might entail transferring patients to a geographically distant specialist service. Furthermore, multiple logics typically exist in the workplace and these must be reconciled (Dodier, 1998). In our case, for example, the logic of deterioration that governs rescue activity may be in tension with the dominant logic of a particular setting, such as the logic of recovery, typical in maternity (Mackintosh and Sandall, 2016) and post-anaesthetic care contexts, with important implications for implementation.

Projects of collective action are mediated through the technologies and materials within a strategic action field. In the example of rescue trajectories, this might include: monitoring equipment, technologies for intervening in care and treatment, and methods for accumulating and displaying vital signs information. Different institutional contexts give rise to distinctive socio-material arrangements and these influence rescue trajectories in significant ways, place different demands on users and shape the distribution of action. Consider, for example, how continuous electronic patient monitoring systems with automated alerts and alarms compare with intermittent manual systems that require vital signs to be measured, recorded and a score calculated by the bedside carer to determine the level of risk and signal a warning alert. Technological differences of this kind have important implications for any improvement effort.

Finally, TMT directs attention to the interpretative repertoires within a strategic action field; that is, the cognitive and meaning making resources available for creating and making sense of objects of practice. There is a growing use of early warning scores and track and trigger tools in rescue trajectories. Track and trigger tools consist of sequential physiological, clinical and observational data recorded and accumulated on a chart (either paper or electronic). When a certain score (achieved by weighting and aggregating each observation) or trigger (observations moving beyond pre-specified parameters) is reached then an escalation response is initiated (Roland *et al.*, 2014). While at one level trigger tools mitigate the risks of task delegation and support team communication, they may also have the unintended consequence of diminishing the importance of other interpretative repertoires within the field – professional pattern recognition and expert patient or carer knowledge – in detecting deterioration. More recent work is concerned with how this “tacit knowledge” can be harnessed effectively, for example, by including staff, patient and family concerns in activation criteria.

### Mechanisms

The final component of TMT is concerned with the mechanisms through which projects of collective action are mobilized. Object formation focuses on how actors draw on the interpretative resources available to them within a strategic action field to create the objects of their practice. Enrollment into an escalation trajectory requires multiple examples of object formation beginning with construction of an individual as at risk of deterioration and a regime of vital signs monitoring instigated, through recognition that the patient’s physiological status is a cause for concern, to the identification of the patient as requiring a specific intervention. How this is achieved is highly dependent on the features of the local strategic action field.

Even when deterioration is identified, marshaling action across professional boundaries/hierarchies can be challenging with differences in language between doctors and nurses and power dynamics contributory factors (Mackintosh and Sandall, 2010). This leads to our next mechanism of mobilization: translation. Derived from ANT (Latour, 2005), in TMT, translation refers to the processes that enable practice objects to be shared and different understandings accommodated. Returning to our example, it points to the actions necessary in order for a patient that is an object of concern for nursing staff to be translated into a clinical priority for the doctor and, if necessary, to be translated into the focus of intervention by the emergency response team. In some contexts with stable teams and strong inter-professional working, translational processes may be accomplished through everyday mechanisms of communication. However, in other cases, the use of structured communication tools – early warning score, SBAR[1] – can be more effective. By transforming a series of discrete observations into a summative indicator of deterioration, tools package the patient’s status into a form that can be readily communicated (Andrews and Waterman, 2005) and can be effective in over-coming professional hierarchies and cultural differences.

The third mechanism in TMT is articulation. Originally developed by Strauss *et al.* (1985), articulation refers to the secondary work processes that align the actions, knowledge and resources necessary for the mobilization of projects of collective action. It is the work that makes the work, work. Responding to deterioration is time critical and articulation work is necessary to ensure the availability of resources and materials to support clinical management. This is not a mundane observation; catastrophic failures in patient safety are often attributed to the lack of functioning equipment (National Patient Safety Agency, 2007) and the absence of monitoring equipment has been identified as a factor undermining the implementation of early warning track and trigger tools (Cioffi, 2000). Attending to articulation in rescue trajectories also underlines the temporal ordering of action and the mechanisms required to achieve this, directing improvement efforts toward the organization’s escalation policy, for example.

The mobilization of rescue trajectories also hinges on staff awareness of at risk patients and contingency planning. Derived from NPT (May and Finch, 2009), reflexive monitoring refers to the processes through which actors collectively or individually appraise and review activity. In a distributed field of action, reflexive monitoring is the means through which members accomplish situational awareness (Gilson, 1995) of an overall project. The importance of situation awareness in rescue trajectories is well recognized, but achieving this is challenging. There is a growing interest in the use of formal mechanisms such as safety huddles and dedicated roles to enable at risk individuals to be identified and contingencies planned for (Brady *et al.*, 2013). Reflexive monitoring is conditioned by the wider institutional context which will include a multiplicity of informal and formal mechanisms designed for this purpose: nursing and medical handovers, the ward round, safety briefings. The form, frequency and effectiveness of these processes in supporting detecting and acting on deterioration would need to be taken into account in any improvement initiative.

The final mechanism in TMT is sensemaking. Derived from the work of the social psychologist Karl Weick (1979, 1995), sensemaking refers to the processes through which agents create order in conditions of complexity. It draws attention to how the material and discursive processes by which members organize their work, account for their actions, and construct the objects of their practice also give meaning and substance to the institutional components of strategic action fields that shape activity and condition future activity. Thus, the structures and maxims through which the ordering of projects is achieved are themselves in a continuous state of becoming as a result of these processes. This draws attention to the dynamic relationship between stability and fluidity in organizations, which for quality improvement purposes has important implications for intervention adaptability, normalization processes, sustainability and organizational learning.

Considered through the lens of TMT, rescue trajectories can be understood as a constellation of socio-material practices and relationships conditioned by the structures and normative resources afforded by the local organizational context (strategic action fields). TMT provides a framework in which these singular arrangements can be methodically examined and the conditions necessary for embedding improvements assessed. It does this because it characterizes and explains the mechanisms through which participants in a strategic action field are enrolled in goal-oriented projects of social action, construct institutional identities for the objects of their practice to accomplish their movement through time and space and, in so doing, perform and produce the institutions in which they are reflexively enrolled. These precepts are summarized in Box II.

## Discussion

This paper has outlined the core elements of TMT, considered its implications for understanding healthcare work and illustrated how it provides a framework for the systematic analysis of collaborative action in different organizational contexts. Table II shows how TMT might be operationalized and is expressed in a series of questions that could be modified for different purposes. The following section considers its wider potential for quality improvement purposes.

Box II **Precepts of translational mobilization theory**

1. Collective, goal-oriented action in institutional settings is mobilized through projects which have contingent outcomes.
2. A project is an institutionally sanctioned socio-technical network of distributed action and actors that follows a trajectory through time and space.
3. Projects are generated by, and generative of, strategic action fields.
4. Strategic action fields are located in institutional contexts, which create the resources that enable, and the conditions that shape, project mobilization.
5. Projects in complex social systems are mobilized through the mechanisms of object formation, articulation, translation, reflexive monitoring and sensemaking.
6. The mechanisms of project mobilization connect the domains of practice and the domains of organization through processes of sensemaking.
7. There is a reciprocal relationship between the production and reproduction of institutionally sanctioned agency and the production and reproduction of institutionally framed objects.

**Source:** Allen and May (2017)

### Mapping the work as it is

Any quality improvement initiative should be founded on an understanding of the processes it is designed to improve. Yet while process mapping is widely used in healthcare, this can fail to capture the work as it is (Waring *et al.*, 2006, Waring and Bishop, 2010). Many practices targeted by quality improvement interventions are poorly understood; workers themselves may not be aware of their contribution to an overall activity (Nardi and Engeström, 1999) and/or feel obliged to offer official rather than realist accounts of how the work is done (Dourish, 2001). TMT offers a framework for understanding service processes that is grounded in the material and cognitive processes through which healthcare activities are accomplished and the relational mechanisms that support or inhibit concerted action whether these are formal or informal (Allen, 2016).

### Taking artifacts seriously

In bringing socio-material relationship center stage, TMT emphasizes the role of artifacts in healthcare practices and acknowledges these as actors that do things in healthcare processes. In so doing it directs attention to their “affordances” (Hutchins, 1995; Gibson, 1979), that is the possibilities they offer for action, the assumptions they embody about the world in which they are used (Latour, 1991) and the preconditions for their use. Despite the calls from science and technology studies for artifacts to be taken seriously in understanding the organization of work, they are rarely adequately conceptualized in quality improvement projects (Allen, 2016, 2017). Unsurprisingly, then, the literature is rich with accounts of the disruptive effects of new technologies (Munkvold *et al.*, 2006) and the impact of the saturation of healthcare systems with mundane technologies such as check lists (Redley and Raggatt, 2017). TMT invites improvers to take seriously the role of artifacts in organizational processes and the local socio-material infrastructures in which they are located (Allen, 2017).

### Connecting content, context and implementation

By connecting the domains of projects, practice and organization and providing a framework for understanding movement between them, TMT also has the potential to contribute to understanding of the inter-relationships between content, context and implementation in improvement. This is necessary to move on from the predictable lists of factors consequential for implementation expressed at such a high level of abstraction they are of little value to those on the ground (Bate, 2013). TMT has particular value in informing functions-focused improvement (Hawe *et al.*, 2009). Here designers of an intervention give practitioners maximal information on the goal to be achieved, but minimal specifications of how to get there. Because TMT is concerned with analyzing the relationships between project elements and their mechanisms of action in the local context, it supports the design of bottom up improvement initiatives of this kind. Relatedly, TMT enables teams systematically to consider barriers and facilitators to change and potential sources of drift (Berg, 1997) arising from the practical activity of the workplace. By bringing to the fore the wider ecology of projects of action – sub-projects, intersecting projects and lines of work – TMT helps to identify and plan for the potential impact of other changes in the system. Finally, as a practice-based approach, TMT shares the domain assumptions and action orientation of normalization process theory (May and Finch, 2009; May *et al.*, 2016), which opens up the potential for these theories to be combined in improvement efforts from problem diagnosis, through intervention development, implementation, sustainability and spread.

### Program theory development

TMT is a middle-range theory like that described by Robert Merton (1949). Middle-range theories bridge the gap between the necessary working hypotheses of empirical studies and the systematic efforts to develop grand theory that will explain all examples of uniform phenomena. Middle-range theories can be used for improvement purposes or combined with informal theories derived from the practice setting, to develop program theories. Program theories have particular value for improvement purposes, typically combining formal and informal theories to frame problem diagnosis, and the design, implementation and evaluation of interventions (Davidoff *et al.*, 2014). TMT offers a useful starting point for such conversations, not least because it focuses on the material and cognitive processes through which healthcare activities are accomplished in context and with which clinicians are familiar, and provides a language to describe these relationships.

### Cross-sector learning

This paper has illustrated the application of TMT to healthcare. But as a generic sociological theory TMT explains and describes the mobilization of emergent projects of collective action wherever these are found, enabling studies of all kinds to be linked together, not by methodology, but by theoretical constructs. This opens up the very real prospect of cross-sector learning and a rebalancing of knowledge sharing between healthcare and other areas. For example, recent international efforts to strengthen service organization typically have involved solutions-driven approaches to improvement, involving the implementation of techniques and approaches derived from systems engineering and management science (Allen *et al.*, 2016). The success of these interventions has been mixed often because of a poor understanding of what an intervention consists of and has often produced “cargo cult improvement,” which like “cargo cult science” (Feynman, 1998), achieves the outward appearance of quality but fails to yield meaningful results because it lacks the essential mechanisms and ingredients. While the importation of improvement technologies from other contexts into healthcare should not be approached uncritically, rather than throwing the proverbial baby out with the bath water, TMT provides a platform for consideration of how such approaches might be modified for use in healthcare and which in turn could extend their reach to other similar organizational contexts.

## Conclusion

This paper introduces TMT and argues that it has potential value for quality improvement purposes in healthcare. TMT is a new theory that addresses an important gap in our frameworks of understanding and has several potential applications. While it invites a radical reframing of healthcare work, in other fields, there are numerous examples of how starting from somewhere else can bring new insights (Collini, 2012) and, as Davidoff *et al.* (2014) argue, there is an urgent need for the use of more formal theory in improvement, not least because it facilitates learning, accumulative understanding and knowledge transfer.

## Figures and Tables

**Table I:** Core components of translational mobilization theory

Core component	Definition	Subcomponent	Definition
Project	An institutionally sanctioned socio-material network of time-bounded collective action which follows a trajectory in time and space	Primary project	The focus of collective action
		Sub-project	A discrete component of collective action within a primary project
		Project actor	Discrete social or material element within a project of action
		Intersecting project	Project of action that may affect or be affected by the primary project
		Line of work	Recurrent activity that feed into multiple projects – such as the work of the X-ray department, the flow of patients through a department or hospital ward, or the caseload of an individual healthcare professional
Strategic action field	The institutional context in which projects emerge and are progressed and which provide the normative and relational frame for collective action	Organizing logics	Elements of a strategic action field that provide a set of normative conventions that define the purpose and scope of possible action
		Structures	Elements of a strategic action field that differentiate social actors (divisions of labor, social worlds, hierarchies, departments, units, teams, interfaces)
		Materials/technologies	Elements of a strategic action field that provide agents with the materials and technologies to support their practice.
		Interpretative repertoires	Elements of a strategic action field that provide agents with a set of cognitive artifacts and relational resources for interpreting and making sense of the objects of practice (classifications, scripts, categories, discourses)
Mechanisms of mobilization and institutionalization	Processes through which agents operating within a strategic action field mobilize projects, drive action and enact institutions	Object formation	Practices that configure the objects of knowledge and practice and enroll them into a network of collective action
		Reflexive monitoring	Practices through which actors evaluate a field of action to generate awareness of project trajectories
		Articulation work	Practices that assemble and align the diverse elements (people, knowledge, materials, technologies, bodies) through which object trajectories and projects of collective action are mobilized Temporal articulation aims to ensure that things happen at the right time and in the right order (Bardram, 2000). Material articulation attends to the availability of resources and materials to support activity (Allen, 2015). Integrative articulation focuses on the coherence of project work (Allen, 2015)
		Translation	Practices that enable practice objects to be shared and differing viewpoints, local contingencies, and multiple interests to be accommodated in order to enable concerted action
		Sensemaking	Practices though which actors order, construct, and mobilize projects and enact structures and institutions

**Source:** Developed from Allen and May (2017)

**Table II:** Operationalizing TMT for quality improvement

Core component	Concepts	Operationalization
Project	Primary project	What is the primary project? (e.g. patient pathway, clinical procedure, organizational process). What is its overarching goal?
	Sub-projects	What are the sub-projects? What are sub-project goals? What are the relationships between sub-projects in the primary project?
	Project actor	What is the focal actor (person, technology, tool, policy) with which you are concerned? What is the function of the project actor within the collective activity? How and in what ways is this linked into the wider network relationships within the project? What are the preconditions for its effectiveness?
	Intersecting projects	Which intersecting projects affect or are affected by the primary project and should be taken into account?
	Lines of work	Which lines of work (department, caseload) should be taken into account? Where are the invisible queues or potential bottlenecks that have implications for project mobilization?
Strategic action field	Organizing logics	What are the organizing logics that drive action in the project? (e.g. triage/prioritization, diagnosis, safety, end of life care, organizational efficiency, rehabilitation). How are these organized in time and space? In what ways are organizing logics congruent or conflicting? How are accommodations achieved between logics?
	Structures	What organizations, departments, teams, professions are involved? How is project work distributed? Who/what are the primary actors? Where is power located? How is the project distributed across time and space? What are the project timescales? Where do project activities take place? Which are critical junctures and dependency relationships between actors and actions? What are the key interfaces between collaborators? What are the modes/mechanisms of communication? (e.g. meetings, events, technologies)
	Materials and technologies	What technologies and materials are involved in the project? How do these condition the possibilities for action? How are these organized in time and space? When are they required? Where are they located? What is involved in maintenance? What information and knowledge sources are involved in the project? How reliable, accessible and comprehensive are the information sources? Who is responsible for generating the information sources?
	Interpretative repertoires	What artifacts and sensemaking resources are involved in the project? (e.g. policies, guidelines, pathways, diagnostic categories). How do these impacts on practice? What is their relationship with the organizing logics involved in the primary or sub-projects and related lines of work?
Mechanisms	Object formation	What are the moments of object formation? Who are the agents involved? What is the purpose of their practice? What artifacts are involved? What are the objects of practice that emerge from these processes? What are their inter-relationships? How are objects of practice distributed in time and space?
	Reflexive monitoring	What are the formal and informal mechanisms of reflexive monitoring? What materials, technologies, and interpretative resources are involved? How intense are reflexive monitoring processes? How is reflexive monitoring work distributed?
	Articulation	What kinds of articulation are required by the project (temporal spatial, material, integrative)? What are the organizing logics that drive articulation work? How is articulation work distributed? Who does the work? When does it take place? Is this formal or informal? Where are the points of disarticulation? What are the materials, technologies and interpretative repertoires that support articulation work?
	Translation	What are the perspective taking and perspective making processes that need to take place in order to collective action to proceed? When are the stabilization moments? Who is responsible? How is this achieved? Where are the critical interfaces between structures and actors in collective action? Where do transfers of care occur? What needs to happen in order for this to be achieved?
	Sensemaking	What are the sensemaking mechanisms involved in project work? When does sensemaking occur? What are the interpretative resources that are drawn upon? Who does this work? What are the roles and responsibilities involved in this work?

## Note

1. SBAR is an acronym for situation, background, assessment, recommendation, a structured communication technique that provides a framework for communicating in healthcare.
